# Effects of Integrated Lumbar Canal Decompression Exercises Program on Pain, Functional Mobility, and Walking Performance in Participants With Lumbar Canal Stenosis

**DOI:** 10.7759/cureus.117219

**Published:** 2026-09-30

**Authors:** Siddhi P Patrekar, Sandeep Shinde, Akshaya V Joshi, Mebin S Thomas

**Affiliations:** 1 Department of Musculoskeletal Sciences, Krishna College of Physiotherapy, Krishna Vishwa Vidyapeeth (Deemed to be University), Karad, IND

**Keywords:** decompression exercises, functional mobility, lumbar canal stenosis, neurogenic claudication, oswestry disability index, pain, self paced walking test, shober's test, tug test, walking performance

## Abstract

Background

Lumbar canal stenosis (LCS) is a common degenerative spinal disorder characterized by narrowing of the lumbar spinal canal, resulting in neural compression, pain, neurogenic claudication, impaired mobility, reduced walking capacity, and functional disability. Conservative physiotherapy interventions are frequently recommended to alleviate symptoms and improve functional outcomes. However, evidence regarding the effectiveness of integrated lumbar canal decompression exercises remains limited. Therefore, this study aimed to compare the effects of an Integrated Lumbar Canal Decompression Exercise Program with a conventional exercise program on pain, functional mobility, walking performance, and disability in individuals with LCS.

Methodology

A comparative study was conducted among 130 participants diagnosed with LCS. Participants were recruited using a simple random sampling technique and allocated into two groups. Group A (control group) received a conventional exercise program, while Group B (experimental group) received an Integrated Lumbar Canal Decompression Exercise Program. Outcome measures included the Numeric Pain Rating Scale (NPRS), Modified Schober Test, Timed Up and Go Test, Self-Paced Walking Test, and Oswestry Disability Index (ODI). Assessments were performed before and after the intervention period. Statistical analysis was conducted using paired and independent t-tests, with a significance level set at p<0.05.

Results

The experimental group demonstrated significant improvements in NPRS at rest (p=0.0413), NPRS during activity (p=0.0365), Modified Schober Test (p=0.0334), Self-Paced Walking Test (p=0.0299), and Oswestry Disability Index (p=0.0227). No significant improvement was observed in the Timed Up and Go Test (p=0.8473). The control group showed significant improvement only in NPRS during activity (p=0.0407) and Oswestry Disability Index (p=0.0209). Between-group analysis revealed significant differences in the Modified Schober Test (p=0.0262) and the ODI (p=0.0119), favoring the experimental group. In contrast, no significant differences were found for pain intensity, the Timed Up and Go Test, or the Self-Paced Walking Test.

Conclusion

Both interventions contributed to improvements in pain and functional outcomes; however, the Integrated Lumbar Canal Decompression Exercise Program demonstrated greater improvements in lumbar mobility and disability between groups. These findings suggest that integrated decompression strategies may have potential as an adjunct to conventional physiotherapy rehabilitation for individuals with LCS. Further randomized, multicentre, and longer-term studies are warranted to confirm these findings and determine their clinical applicability.

## Introduction

Lumbar canal stenosis (LCS) is a degenerative spinal disorder characterized by narrowing of the spinal canal, which may result in compression of the neural and vascular structures within the lumbar spine. It commonly affects middle-aged and older adults and is an important cause of pain, disability, and reduced mobility in the elderly population. The pathophysiology of LCS involves progressive degenerative changes, including intervertebral disc degeneration, facet joint hypertrophy, ligamentum flavum thickening, and osteophyte formation, which contribute to a reduction in the available space for neural tissues and may lead to neurological symptoms [1].

LCS has been recognized as a significant cause of functional impairment and reduced quality of life. Clinical manifestations include low back pain, radiating leg pain, numbness, weakness, and activity limitations that progressively affect daily living [2]. Recent literature has emphasized the multifactorial nature of lumbar stenosis and highlighted the importance of accurate diagnosis and appropriate conservative management strategies [3]. Back and leg pain are among the most common presenting complaints and often lead to reduced physical activity and functional independence [4].

The severity of LCS has been shown to influence walking ability and functional performance. Individuals with greater stenotic changes frequently demonstrate impaired gait, reduced walking endurance, and decreased participation in daily activities [5]. A hallmark symptom of LCS is neurogenic claudication, which is characterized by pain, numbness, tingling, or weakness in the lower extremities that worsens with walking or prolonged standing and is relieved by sitting or lumbar flexion [6].

Conservative treatment remains the first-line approach for many individuals with LCS and includes physiotherapy interventions aimed at reducing symptoms and improving function. Non-operative management strategies such as exercise therapy, manual therapy, traction, and patient education have demonstrated beneficial effects on pain and functional outcomes [7]. Functional mobility is commonly assessed using the Timed Up and Go Test, which provides valuable information regarding walking capacity, mobility, balance, and activities of daily living in individuals with lumbar spinal disorders [8]. In addition, balance impairments, pain severity, and quality of life have been identified as important factors influencing functional status in patients with LCS [9].

Walking limitation is one of the most disabling consequences of LCS. The Self-Paced Walking Test is frequently used to evaluate walking tolerance and functional walking capacity in the affected individuals [10]. Comprehensive nonsurgical rehabilitation programs have demonstrated effectiveness in improving walking performance and overall functional outcomes [11]. Furthermore, self-reported walking capacity has been shown to correlate with clinical findings and symptom severity in patients with lumbar stenosis [12].

An assessment of lumbar mobility is also important when evaluating treatment outcomes. The Modified Schober Test is a reliable clinical measure used to assess lumbar flexion and spinal mobility, and exercise interventions have been shown to improve these parameters while reducing pain and disability [13]. Recent advances in conservative management have explored the use of segmental traction therapy, which may facilitate spinal decompression and improve symptoms through mechanical unloading of lumbar structures [14]. Previous studies have further demonstrated that the addition of segmental traction to routine physiotherapy may enhance pain relief and functional recovery [15].

More recently, the Modified Reversed Contralateral Axial Rotation (MRCAR) position has been proposed as a decompression-oriented intervention designed to reduce nerve root compression and improve spinal alignment. Preliminary findings suggest that this technique may provide symptomatic relief and functional benefits in individuals with lumbar spine disorders [16]. A systematic review of physiotherapy interventions for degenerative lumbar spinal stenosis highlighted the effectiveness of multimodal rehabilitation approaches incorporating exercise therapy, stabilization exercises, traction techniques, and patient education [17,18].

Therefore, the present study was undertaken to compare the effectiveness of an Integrated Lumbar Canal Decompression Exercise Program and a conventional exercise program on pain, lumbar mobility, functional mobility, walking performance, and disability in individuals with LCS.

## Materials and methods

Study design

A comparative study was conducted to evaluate the effects of an Integrated Lumbar Canal Decompression Exercise Program compared with a conventional exercise program in individuals with LCS. Pain intensity and disability were considered the primary outcomes, while lumbar mobility, functional mobility, and walking performance were considered the secondary outcomes. Treatment effects were evaluated using between-group effect sizes with 95% confidence intervals (CIs), along with p-values, to assess the magnitude and precision of the intervention effects.

Sample size

The sample size was estimated using a prevalence-based sample-size formula:

$n = \frac{Z^2 \times p \times q}{d^2}$

The prevalence of lumbar spinal stenosis reported by Jensen et al. ranged from 11% in the general population to 25%-39% in clinical populations. Using a 95% confidence level (Z=1.96), a prevalence (p) of 11% (0.11), q=1−p (0.89), and an allowable error (d) of 5% (0.05), the estimated sample size was approximately 150 participants.

However, this calculation was based on disease prevalence and was not based on an a priori statistical power analysis. Considering participant availability and the predefined inclusion and exclusion criteria, 130 eligible participants were recruited and completed the study. All 130 participants completed the required assessments and intervention protocol and were included in the final analysis. No post-hoc power analysis was performed. The difference between the calculated prevalence-based sample size and the final sample size is acknowledged as a limitation of the study.

Sampling technique

The study was conducted following approval from the Institutional Ethics Committee of Krishna Vishwa Vidyapeeth, Deemed to be University, Karad (Ethics Committee Approval No.291/2025-2026), dated November 5, 2025. Participants were recruited from Krishna Hospital, Karad. Written informed consent was obtained from all participants before enrollment in the study. Participants were recruited using a simple random sampling technique from the outpatient physiotherapy and orthopedic departments. Individuals diagnosed with lumbar canal stenosis were screened according to the predefined inclusion and exclusion criteria. After eligibility was confirmed, written informed consent was obtained before enrollment. Eligible participants were then randomly allocated into two groups using a simple lottery method (chit method). Each participant was assigned a group by drawing a concealed chit indicating either Group A or Group B. Group A received the conventional exercise program, while Group B received the Integrated Lumbar Canal Decompression Exercise Program.

Inclusion criteria

Participants were selected according to predefined inclusion and exclusion criteria. Individuals aged between 40 and 80 years, including both men and women, with radiating pain in one or both lower limbs and symptoms of neurogenic intermittent claudication were included in the study. Participants were required to have a Body Mass Index (BMI) of less than 35 kg/m² and a confirmed diagnosis of lumbar canal stenosis based on magnetic resonance imaging (MRI) findings.

Exclusion criteria

Individuals were excluded if they had a history of spinal surgery, spinal deformities, vertebral fractures, systemic inflammatory disorders, or pregnancy. Participants with uncontrolled medical conditions that could interfere with exercise participation or influence study outcomes, such as hypertension, diabetes mellitus, and thyroid disorders, were also excluded from the study.

Statistical analysis

Statistical analysis was performed using IBM SPSS Statistics for Windows, Version 29.0 (IBM Corp., Armonk, NY, USA; 2023). Descriptive statistics were used to summarize demographic and clinical characteristics and are presented as mean±standard deviation (SD) for continuous variables and frequencies and percentages for categorical variables. The normality of continuous outcome variables was assessed using the Shapiro-Wilk test. The data were considered normally distributed when the Shapiro-Wilk test was non-significant (p>0.05). Based on the assessment of normality, parametric statistical tests were used. Within-group comparisons between pre- and post-intervention measurements were performed using paired-samples t-tests, while between-group comparisons were performed using independent-samples t-tests. The outcome measures included the Numeric Pain Rating Scale (NPRS), Modified Schober Test, Timed Up and Go Test, Self-Paced Walking Test, and Oswestry Disability Index (ODI). Effect sizes with 95% CI were also reported to quantify the magnitude and precision of the treatment effects. A two-tailed p-value of <0.05 was considered statistically significant.

Procedure

The study was conducted after obtaining approval from the Institutional Ethics Committee of Krishna Vishwa Vidyapeeth (Deemed to be University), Karad. The Institutional Ethics Committee approved the initiation of the research project under approval number 291/2025-2026. All participants were informed about the study procedures, and written informed consent was obtained prior to participation. Participants diagnosed with LCS were screened according to the inclusion and exclusion criteria. Eligible participants were informed about the purpose and procedure of the study, and written informed consent was obtained before participation. Baseline assessments were conducted using the Numeric Pain Rating Scale (NPRS), Modified Schober Test, Timed Up and Go Test, Self-Paced Walking Test, and ODI.

A total of 130 participants were recruited and allocated into two groups. Group A (Control Group) received a conventional exercise program, while Group B (Experimental Group) received an Integrated Lumbar Canal Decompression Exercise Program. The conventional exercise program consisted of lumbar flexion exercises, stretching exercises, strengthening exercises, and routine physiotherapy interventions. The Integrated Lumbar Canal Decompression Exercise Program included decompression-oriented exercises, segmental traction therapy, Modified Reversed Contralateral Axial Rotation (MRCAR) positioning, lumbar flexion exercises, stretching exercises, and strengthening exercises.

Both groups received their respective interventions according to the prescribed treatment protocol. Following the completion of the intervention period, all outcome measures were reassessed using the same assessment tools. The collected data were recorded and analyzed to compare the effectiveness of the two intervention programs on pain, lumbar mobility, functional mobility, walking performance, and disability in individuals with lumbar canal stenosis.

The study flowchart illustrates the recruitment, screening, allocation, intervention, and outcome assessment procedures of participants with lumbar canal stenosis. A total of 130 participants were allocated into either the control group or the experimental group and underwent an eight-week intervention program followed by post-intervention assessment and statistical analysis, as given in Figure 1.

**Figure 1:**
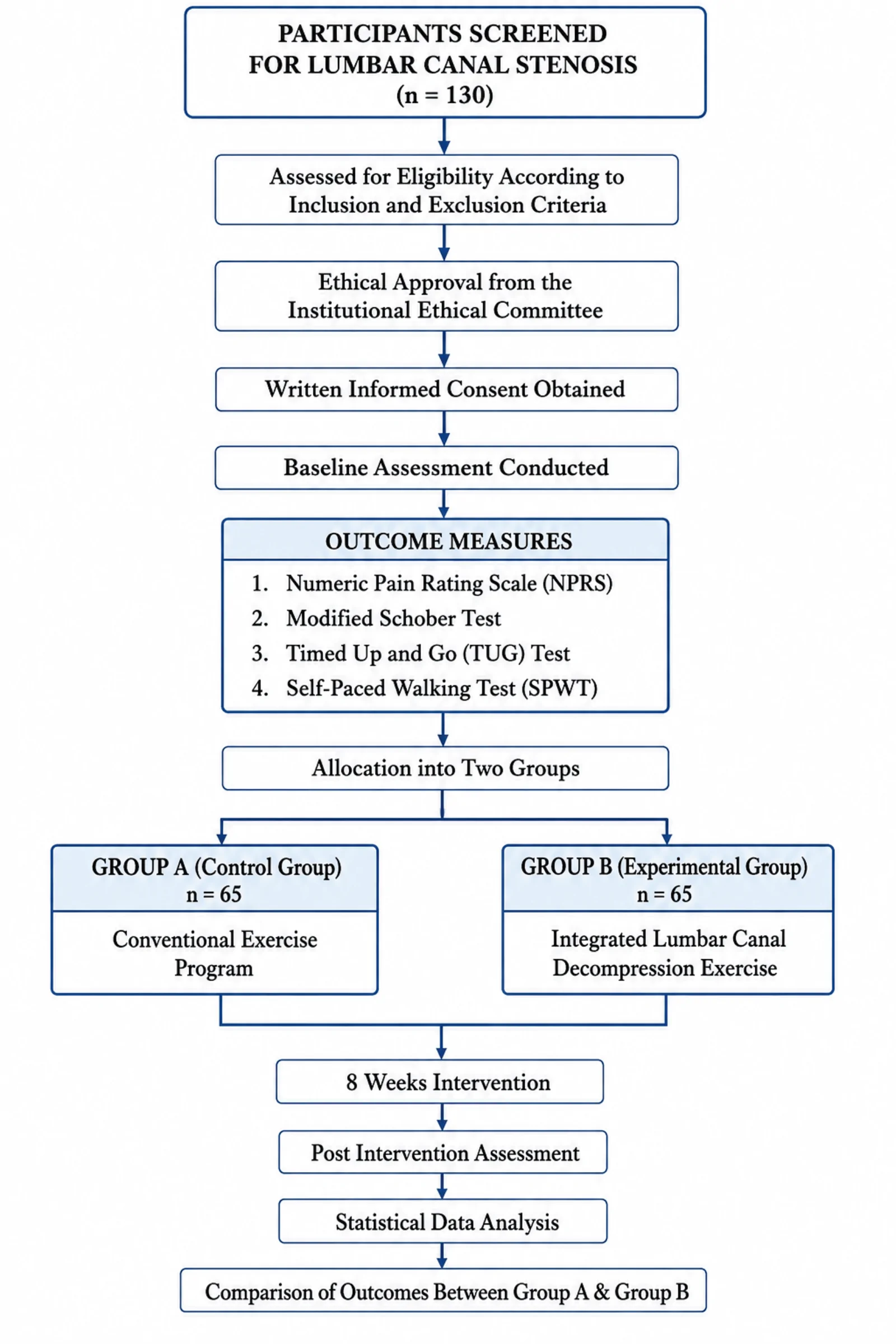
Study flowchart illustrating the recruitment, screening, allocation, intervention, and outcome assessment procedures of participants with lumbar canal stenosis. Flowchart illustrating participant screening, eligibility assessment, allocation, intervention, outcome assessment, and statistical analysis. Created using Microsoft PowerPoint (Microsoft Corporation, Redmond, WA, USA).

Outcome measure

Numeric Pain Rating Scale

NPRS is a valid and reliable self-reported measure used to assess pain intensity. It consists of an 11-point numerical scale ranging from 0 to 10, where 0 indicates no pain and 10 represents the worst imaginable pain. Participants are asked to rate their current pain intensity by selecting the number that best describes their pain. The NPRS is simple, easy to administer, and widely used in clinical practice and research to evaluate pain severity and monitor treatment outcomes [19].

Modified Schober Test

The Modified Schober Test is a simple and reliable clinical measure used to assess lumbar spine flexibility and range of motion, particularly lumbar flexion. During the test, a mark is made at the level of the posterior superior iliac spine (PSIS), and additional marks are placed 5 cm below and 10 cm above this point. The participant is then asked to bend forward maximally while keeping the knees straight. The increase in the distance between the marks is measured in centimeters. A greater increase in distance indicates better lumbar spine mobility, whereas a reduced increase suggests restricted lumbar flexion. The Modified Schober Test is widely used in clinical practice and research to evaluate spinal mobility and monitor treatment outcomes in patients with lumbar spine disorders [20].

Timed Up and Go Test

The Timed Up and Go Test is a simple, reliable, and widely used clinical assessment tool for evaluating functional mobility, balance, gait performance, and fall risk. During the test, the participant is instructed to rise from a standard chair, walk a distance of 3 meters at a comfortable and safe pace, turn around, walk back to the chair, and sit down. The total time taken to complete the task is recorded in seconds. Lower completion times indicate better functional mobility and balance, whereas longer times suggest impaired mobility and an increased risk of falls. The Timed Up and Go Test is commonly used in individuals with musculoskeletal, neurological, and geriatric conditions and is considered a valuable outcome measure for monitoring treatment effectiveness [21].

Self-Paced Walking Test

The Self-Paced Walking Test is a valid and reliable functional performance measure used to assess walking capacity and walking tolerance in individuals with lumbar spinal stenosis and neurogenic claudication. During the test, participants are instructed to walk at their normal, comfortable pace on a predetermined course until symptoms such as leg pain, numbness, weakness, or fatigue prevent them from continuing. The total walking distance and/or walking time are recorded. Greater walking distance and longer walking duration indicate better functional walking ability, whereas reduced performance reflects limitations associated with neurogenic claudication and impaired mobility. The Self-Paced Walking Test is widely used in clinical practice and research to evaluate treatment outcomes and functional improvement in patients with lumbar canal stenosis [22].

Oswestry Disability Index

ODI is a standardized, valid, and reliable self-reported questionnaire used to assess the degree of disability and functional impairment associated with low back pain. The questionnaire consists of 10 sections that evaluate various activities of daily living, including pain intensity, personal care, lifting, walking, sitting, standing, sleeping, social life, traveling, and employment or homemaking activities. Each section is scored from 0 to 5, with higher scores indicating greater disability. The total score is expressed as a percentage, ranging from 0% (no disability) to 100% (maximum disability). The ODI is widely regarded as the gold standard outcome measure for evaluating functional disability and treatment outcomes in patients with lumbar spine disorders [23].

Physiotherapy intervention

Both groups underwent an eight-week progressive physiotherapy intervention. The exercise program was progressed according to the treatment phase, with exercises targeting breathing, abdominal stabilization, lower-limb strengthening, lumbar mobility, core strengthening, canal widening, and lumbar stabilization.

During Weeks 1-2, both groups performed deep breathing exercises, abdominal drawing-in maneuvers, isometric hip flexor, extensor, adductor, and abductor strengthening, and lower-limb range-of-motion exercises. During Weeks 2-3, stabilization exercises (Levels 1-3), including supine pelvic tilt, half sit-ups, cat-and-camel, and adduction lifts, were introduced. During Weeks 4-6, the program progressed to forward bending, single-knee-to-chest exercises, stabilization exercises (Levels 4-6), prone lumbar and gluteal extension, curl-ups, and diagonal curl-ups. During Weeks 7-8, participants performed double-knee-to-chest, child’s pose, bilateral straight-leg raise, and lumbar flexion self-stretching exercises. The prescribed dosage consisted primarily of 10 repetitions × one set for dynamic exercises and 10-second holds × 10 repetitions × one set for specified holding exercises. Isometric hip strengthening was performed using 10-second holds × five repetitions × one set. The MRCAR position was performed for a 15-second hold × 10 repetitions × one set. 

The principal difference between the groups was the addition of decompression-focused interventions in the experimental group. Group B received the MRCAR position and Segmental Integrated Lumbar Traction, whereas Group A received conventional Integrated Lumbar Traction. Both groups also received Hot Moist Pack for 15 minutes and Interferential Therapy for 15 minutes throughout the intervention. Lumbar traction was administered for 30 minutes in both groups, with segmental integrated lumbar traction used in the experimental group, as given in Table 1.

**Table 1:** Week-wise rehabilitation program for participants with lumbar canal stenosis HMP=Hot Moist Pack; IFT=Interferential Therapy; MRCAR=Modified Reversed Contralateral Axial Rotation; reps=repetitions; sec=seconds; min=minutes.

Week	Exercise/Intervention	Control Group	Experimental Group
1-2	Deep Breathing Exercises	10 reps × 1 set	10 reps × 1 set
	Abdominal Drawing-In Maneuver	10 reps × 1 set	10 reps × 1 set
	Isometric Hip Flexor/Extensor/Adductor/Abductor	10 sec hold × 5 reps × 1 set	10 sec hold × 5 reps × 1 set
	Straight Leg Raise, Adduction, Abduction, Heel Slides, Ankle Toe Movements	10 reps × 1 set	10 reps × 1 set
2-3	Stabilization Exercise Levels 1-3	10 reps × 1 set	10 reps × 1 set
	Supine Pelvic Tilt, Half Sit-Ups, Cat and Camel, and Adduction Lifts	10 sec hold × 10 reps × 1 set	10 sec hold × 10 reps × 1 set
4-6	Forward Bending, Single Knee-to-Chest	10 sec hold × 10 reps × 1 set	10 sec hold × 10 reps × 1 set
	Stabilization Exercise Levels 4-6	10 reps × 1 set	10 reps × 1 set
	Prone Lumbar and Gluteal Extension, Curl-Ups, Diagonal Curl-Ups	10 sec hold × 10 reps × 1 set	10 sec hold × 10 reps × 1 set
7-8	Double Knee-to-Chest, Child Pose, Bilateral Straight Leg Raise	10 sec hold × 10 reps × 1 set	10 sec hold × 10 reps × 1 set
	Lumbar Flexion Self-Stretching	10 sec hold × 10 reps × 1 set	10 sec hold × 10 reps × 1 set
	MRCAR Position	Not Given	15 sec hold × 10 reps × 1 set
	Lumbar Traction	Integrated Lumbar Traction – 30 min	Segmental Integrated Lumbar Traction – 30 min
Throughout	Hot Moist Pack (HMP)	15 min	15 min
	Interferential Therapy (IFT)	15 min	15 min

## Results

A total of 130 participants with LCS were included in the study. The mean age was 55.5±9.05 years, with women (60.76%) outnumbering men (39.24%). The mean BMI was 27.92±2.8 kg/m². Baseline assessment showed moderate pain levels at rest (NPRS: 3.97±1.93) and during activity (5.66±2.11), reduced lumbar mobility (Modified Schober's Test: 3.71±0.45 cm), impaired functional mobility (Timed Up and Go Test: 14.07±2.86 seconds), limited walking capacity (Self-Paced Walking Test: 285.60±57.98 m), and moderate disability (ODI: 44.56±10.74), indicating significant functional limitations associated with lumbar canal stenosis, as given in Table 2.

**Table 2:** Demographic and Baseline Characteristics of the Study Participants (N=130) BMI: Body Mass Index; NPRS: Numeric Pain Rating Scale; TUG: Timed Up and Go Test; ODI: Oswestry Disability Index; SD: Standard Deviation.

Variables	Mean (SD) or Frequency (%)
Age (mean±SD)	55.5±9.05
Sex, n%	
Female	79 (60.76)
Male	51 (39.24)
BMI (mean±SD)	27.92±2.8
NPRS on rest (mean±SD)	3.97±1.93
NPRS on Activity (mean±SD)	5.66±2.11
Shober’s test (mean±SD)	3.71±0.45
Timed Up and Go Test (mean±SD)	14.07±2.86
Self-Paced Walking Test (mean±SD)	285.60±57.98
ODI (mean±SD)	44.56±10.74

A comparison of pre-test and post-test outcomes of Group A following the intervention showed a significant reduction in NPRS during activity (MD=0.91, p=0.0407) and ODI scores (MD=0.66, p=0.0209), indicating improvements in pain and disability. Improvements were also observed in NPRS at rest, Shober's Test, Timed Up and Go Test, and Self-Paced Walking Test; however, these changes were not statistically significant (p>0.05). Overall, the intervention was effective in reducing activity-related pain and disability among participants, as given in Table 3.

**Table 3:** Pre- and Post-Intervention Comparison of Outcome Measures in Group A (Control Group) NPRS: Numeric Pain Rating Scale; SD: Standard Deviation.

Outcome Measures	Pre-test (Mean±SD)	Post-test (Mean±SD)	Mean Difference	P value	T value
NPRS					
At Rest	2.35±1.02	1.64±3.08	0.71	0.0616	1.903
On Activity	3.86±1.14	2.95±3.67	0.91	0.0407	2.089
Shober’s Test	3.78±0.47	4.009±2.263	-0.229	0.4261	0.8011
Timed Up and Go Test	11.86±1.49	11.41±3.62	0.45	0.2849	1.078
Self-Paced Walking Test	325.44±8.7	323.16±29.4	2.28	0.4998	0.6786
Oswestry Disability Index	37.44±4.28	36.78±4.36	0.66	0.0209	2.368

A comparison of pre-test and post-test outcomes in Group B intervention showed significant improvements in NPRS at rest (MD=0.54, p=0.0413), NPRS during activity (MD=1.10, p=0.0365), Shober’s Test (MD=0.57, p=0.0334), Self-Paced Walking Test (MD=23.97, p=0.0299), and ODI (MD=0.72, p=0.0227). However, the improvement in the Timed Up and Go Test was not statistically significant (MD=0.15, p=0.8473). Overall, the intervention demonstrated significant benefits in reducing pain, improving lumbar mobility, walking capacity, and decreasing disability, as given in Table 4.

**Table 4:** Pre- and Post-Intervention Comparison of Outcome Measures in Group B (Experimental Group) NPRS: Numeric Pain Rating Scale; SD: Standard Deviation.

Outcome Measures	Pre-test (Mean±SD)	Post-test (Mean±SD)	Mean Difference	P value	T value
NPRS					
At Rest	2.35±1.02	1.81±2.128	0.54	0.0413	2.082
On Activity	3.96±1.36	2.86±4.17	1.10	0.0365	2.136
Shober’s Test	3.86±0.90	4.43±1.96	-0.57	0.0334	2.174
Timed Up and Go Test	12.07±2.05	11.92±6.17	0.15	0.8473	0.1933
Self-Paced Walking Test	326.83±10.30	302.86±83.46	23.97	0.0299	2.222
Oswestry Disability Index	37.44±4.28	36.72±4.81	0.72	0.0227	2.335

A post-test comparison between Group A and Group B showed a statistically significant difference in Shober’s Test (MD=0.78, p=0.0262) and ODI (MD=0.06, p=0.0119), indicating better improvement in lumbar mobility and disability outcomes between the groups. No significant differences were found for NPRS at rest (p=0.0718), NPRS during activity (p=0.3428), Timed Up and Go Test (p=0.5686), or Self-Paced Walking Test, also improved within the intervention groups; however, the between-group difference did not reach statistical significance (p=0.0666). Therefore, the findings do not support a statistically significant superiority of the integrated program over the conventional exercise program for walking performance, as given in Table 5.

**Table 5:** Between-Group Comparison of Post-Intervention Outcome Measures NPRS: Numeric Pain Rating Scale; SD: Standard Deviation.

Outcome Measures	Post-test values of Group A (Mean±SD)	Post-test values of Group B (Mean±SD)	Mean Difference	P value	T value
NPRS					
At Rest	1.64±3.08	1.81±2.128	-0.53	0.0718	1.815
On Activity	2.95±3.67	2.86±4.17	-0.51	0.3428	0.9522
Shober’s Test	3.95±2.32	4.73±1.59	0.78	0.0262	2.249
Timed Up and Go Test	11.41±3.62	11.92±6.175	-0.51	0.5686	0.5715
Self-Paced Walking Test	323.16±29.41	302.86±83.46	20.30	0.0666	1.850
Oswestry Disability Index	36.78±4.36	36.72±4.810	0.06	0.0119	2.552

## Discussion

The present comparative study evaluated the effectiveness of an Integrated Lumbar Canal Decompression Exercise Program in comparison with a conventional exercise program in individuals with LCS. The findings demonstrated improvements in pain intensity, lumbar mobility, walking performance, and functional disability in both groups; however, greater improvements were observed in the experimental group, particularly in lumbar mobility and disability outcomes. These findings support the role of decompression-oriented physiotherapy interventions in the conservative management of lumbar canal stenosis.

Pain is one of the primary symptoms associated with LCS and may contribute to reduced activity levels and impaired quality of life. In the present study, significant reductions in pain during activity were observed in both groups, while the experimental group also demonstrated significant improvement in pain at rest. These improvements may be related to the combined effects of exercise, traction, and the positioning strategies incorporated into the integrated intervention. A possible mechanism underlying the additional benefit of the integrated approach may involve the combined mechanical effects of segmental traction and the MRCAR position. Standard lumbar traction primarily applies a longitudinal distraction force, whereas MRCAR incorporates lumbar flexion with side bending and axial rotation toward the pain-free side. A recent three-dimensional CT study directly investigated MRCAR and demonstrated an increase in the cross-sectional area of the lumbar intervertebral foramen and an improvement in straight-leg-raising angle in individuals with lumbar disc prolapse and radiculopathy, suggesting a position-specific foraminal decompressive effect. These findings provide anatomical support for the proposed decompressive role of MRCAR; however, they were obtained in patients with lumbar disc prolapse and radiculopathy rather than LCS.

In the present study, no imaging, intradiscal pressure, canal dimensions, foraminal dimensions, or other physiological measurements were obtained. Therefore, it cannot be determined whether MRCAR combined with segmental traction produced greater changes in spinal canal or foraminal dimensions than standard traction alone. A possible mechanism, not directly tested in the present study, might be that the combination of longitudinal distraction with multiplanar positioning provides a different mechanical loading pattern from traction alone and may transiently influence neural or foraminal space. This interpretation should therefore be considered hypothesis-generating rather than a demonstrated physiological mechanism.

The observed clinical improvements may consequently reflect the combined effects of the integrated intervention rather than the isolated effect of MRCAR positioning. Further studies incorporating dynamic MRI or CT, as well as biomechanical or physiological measurements, are required to determine whether MRCAR combined with segmental traction produces measurable changes in canal or foraminal dimensions in individuals with lumbar canal stenosis [6].

Lumbar mobility, assessed using the Modified Schober Test, showed significant improvement in the experimental group and a significant difference between groups at post-test assessment. Restricted lumbar mobility is a common consequence of degenerative spinal changes, pain-related muscle guarding, and altered movement patterns. The improvement observed in the present study may be explained by the incorporation of lumbar flexion exercises, decompression positioning, and stretching interventions that promote increased spinal flexibility. These findings are in agreement with the study conducted by Do and Chon, who demonstrated that flexion-based exercise programs significantly improved lumbar range of motion and functional outcomes in patients with lumbar spinal stenosis [13]. The superior improvement observed in the experimental group suggests that decompression-based interventions may provide additional benefits beyond those achieved through conventional exercise programs alone.

Walking limitation resulting from neurogenic claudication is considered one of the most disabling manifestations of lumbar canal stenosis. In the current study, significant improvement in Self-Paced Walking Test performance was observed within the experimental group, whereas no significant between-group difference was identified. Nevertheless, the magnitude of improvement suggests a clinically meaningful enhancement in walking tolerance. These findings support previous research by Rainville et al., who emphasized the importance of walking performance as an indicator of functional status in individuals with lumbar spinal stenosis [10]. Likewise, Ammendolia et al. demonstrated that comprehensive non-surgical rehabilitation programs can improve walking ability and functional capacity in this patient population. Improved walking performance may result from reduced neural compression, improved trunk stability, enhanced muscular endurance, and decreased pain during ambulation [11].

The Timed Up and Go Test did not demonstrate statistically significant improvement in either group. Although a trend toward improvement was observed, the changes were insufficient to reach statistical significance. This finding may be related to the multifactorial nature of functional mobility, which is influenced not only by pain and spinal mobility but also by balance, muscle strength, age-related factors, and psychological confidence during movement. Stienen et al. reported that Timed Up and Go Test performance is closely associated with overall functional status in patients with lumbar spinal stenosis; however, measurable changes may require longer intervention durations and more intensive balance-oriented training [8]. Therefore, the absence of significant improvement in Timed Up and Go Test performance does not necessarily indicate a lack of clinical benefit but may reflect the complexity of functional mobility recovery in this population.

Functional disability, measured using the ODI, showed significant improvement in both groups, with a statistically significant difference favoring the experimental group. Reduction in disability scores indicates improved ability to perform activities of daily living and enhanced functional independence. These findings are consistent with those reported by Macedo et al., who concluded that physiotherapy interventions can positively influence disability and functional outcomes in individuals with degenerative lumbar spinal stenosis. Similarly, Ammendolia et al. demonstrated that comprehensive rehabilitation approaches are associated with meaningful improvements in disability and quality of life [17]. The greater improvement observed in the experimental group suggests that combining decompression-based techniques with conventional therapeutic exercises may provide superior functional benefits.

The positive outcomes observed in the experimental group may also be explained by the inclusion of segmental traction therapy and the Modified Reversed Contralateral Axial Rotation position. Segmental traction is believed to increase intervertebral space, decrease nerve root compression, and reduce mechanical stress on spinal structures. Karimi et al. and Akberov et al. reported favorable effects of segmental traction on pain reduction and functional improvement in patients with lumbar spine disorders [14,15]. Furthermore, Raffet et al. recently demonstrated that the MRCAR position may facilitate neural decompression by optimizing foraminal dimensions and reducing nerve root impingement [16]. Although the present study did not directly measure anatomical changes, the improvements in pain, mobility, and disability support the potential clinical value of these decompression-oriented interventions.

Overall, the findings of the present study suggest that both conventional physiotherapy and integrated lumbar canal decompression exercises are beneficial for individuals with lumbar canal stenosis. However, the integrated decompression program produced superior improvements in lumbar mobility and functional disability, indicating that addressing neural decompression in addition to general exercise may enhance rehabilitation outcomes. These findings support current evidence advocating comprehensive, conservative management approaches for lumbar canal stenosis and highlight the potential role of decompression-based physiotherapy interventions in optimizing patient recovery.

Strengths

The present study possesses several strengths. First, it employed a comparative design that enabled direct evaluation of an Integrated Lumbar Canal Decompression Exercise Program against a conventional exercise program in individuals with lumbar canal stenosis. Second, multiple validated outcome measures, including the Numeric Pain Rating Scale (NPRS), Modified Schober Test, Timed Up and Go Test, Self-Paced Walking Test, and ODI, were utilized to comprehensively assess pain, lumbar mobility, functional mobility, walking performance, and disability. Third, the study incorporated a relatively large sample size, enhancing the reliability and clinical applicability of the findings. Furthermore, the inclusion of decompression-oriented interventions such as segmental traction and the MRCAR position provided valuable evidence regarding innovative conservative treatment strategies for lumbar canal stenosis.

Limitations

The present study has several limitations that should be considered when interpreting the findings. First, although eligible participants were allocated to the two intervention groups using a simple randomization procedure, the method of randomization was not adequately documented in the original study protocol. Therefore, the possibility of allocation-related bias cannot be completely excluded. Second, blinding of participants and treating physiotherapists was not feasible because of the nature of the exercise interventions, and outcome-assessor blinding was not implemented/reported. This may have introduced performance or assessment bias. Third, the original sample-size estimation was based on a prevalence-based formula rather than an a priori power calculation. Although approximately 150 participants were estimated using the prevalence-based formula, 130 eligible participants completed the study. No post-hoc power analysis was performed; therefore, the possibility that the study was insufficiently powered to detect some between-group differences cannot be excluded. Future studies should use an a priori power calculation based on a clinically meaningful between-group effect and prespecified primary outcome. Fourth, the study did not prespecify a single primary outcome; pain, lumbar mobility, functional mobility, walking performance, and disability were evaluated as study outcomes. This may increase the risk of multiple statistical comparisons and makes interpretation of the overall treatment effect more difficult. Future trials should clearly define a primary outcome before recruitment. Fifth, the comparative study design and relatively short intervention and follow-up period limit the ability to establish definitive causal relationships and determine the long-term sustainability of the observed improvements. Longer follow-up periods and randomized controlled trial designs are required to evaluate the durability of treatment effects. Sixth, the sample was recruited from a single clinical setting, which may limit the generalizability of the findings to broader and more diverse populations. Finally, the study primarily assessed clinical and functional outcomes and did not include radiological or physiological measurements to evaluate structural or biomechanical changes following treatment. Therefore, the proposed mechanisms related to decompression, MRCAR positioning, and changes in neural or foraminal dimensions remain hypothetical and were not directly verified in this study. Future studies incorporating appropriate imaging and physiological measures are warranted to investigate these mechanisms.

Clinical implications

The findings of this study have potential clinical implications for physiotherapy management of individuals with lumbar canal stenosis. The Integrated Lumbar Canal Decompression Exercise Program may be considered as an adjunct to conventional physiotherapy, particularly when the treatment goal is to improve lumbar mobility and reduce functional disability. The program combines conventional therapeutic exercises with decompression-oriented interventions, including the MRCAR position and segmental integrated lumbar traction. The significant improvements observed in lumbar mobility and disability in the experimental group suggest that incorporating these components into a comprehensive rehabilitation program may provide additional clinical benefits. However, because the study evaluated short-term outcomes and was conducted in a single clinical setting, clinicians should consider individual patient characteristics, symptom severity, tolerance to exercise, and clinical response when applying this intervention. Further multicenter studies with larger samples and longer follow-up are warranted before broad clinical recommendations can be made.

Future scope

Future research may explore the long-term effectiveness of Integrated Lumbar Canal Decompression Exercise Programs through extended follow-up periods. Studies incorporating larger multicenter samples may further strengthen the generalizability of findings. The inclusion of advanced imaging techniques such as magnetic resonance imaging (MRI) or ultrasonography may help evaluate structural and biomechanical changes associated with decompression-based interventions. Future studies may also investigate the effectiveness of these interventions in different severity levels of lumbar canal stenosis and compare their outcomes with other conservative and surgical treatment approaches. Additionally, the integration of technology-assisted rehabilitation, tele-rehabilitation, and individualized exercise protocols may further enhance patient outcomes and treatment accessibility.

## Conclusions

The findings of the present study indicate that both conventional physiotherapy and the Integrated Lumbar Canal Decompression Exercise Program were associated with improvements in clinical and functional outcomes in individuals with LCS. However, the Integrated Lumbar Canal Decompression Exercise Program demonstrated statistically significant greater between-group improvements in lumbar mobility, as assessed by the Modified Schober Test, and functional disability, as assessed by the ODI, compared with the conventional exercise program. Although improvements in pain and walking performance were observed following the intervention, the between-group difference in the Self-Paced Walking Test did not reach statistical significance. Therefore, the findings support the potential benefit of the Integrated Lumbar Canal Decompression Exercise Program, particularly for improving lumbar mobility and reducing functional disability, while its effects on other clinical outcomes should be interpreted with consideration of the observed between-group statistical results.
